# Inhibitory effects of two types of food additives on biofilm formation by foodborne pathogens

**DOI:** 10.1002/mbo3.853

**Published:** 2019-06-09

**Authors:** Liyan Liu, Congxiu Ye, Thanapop Soteyome, Xihong Zhao, Jing Xia, Wenyi Xu, Yuzhu Mao, Ruixin Peng, Jinxuan Chen, Zhenbo Xu, Mark E. Shirtliff, Janette M. Harro

**Affiliations:** ^1^ School of Food Science and Engineering, Guangdong Province Key Laboratory for Green Processing of Natural Products and Product Safety South China University of Technology Guangzhou China; ^2^ Department of Dermato‐Venereology Third Affiliated Hospital of Sun Yat‐sen University Guangzhou China; ^3^ Home Economics Technology Rajamangala University of Technology Phra Nakhon Bangkok Thailand; ^4^ Research Center for Environmental Ecology and Engineering, School of Environmental Ecology and Biological Engineering Wuhan Institute of Technology Wuhan China; ^5^ Department of Microbial Pathogenesis, School of Dentistry University of Maryland Baltimore Maryland; ^6^ Overseas Expertise Introduction Center for Discipline Innovation of Food Nutrition and Human Health (111 Center) Guangzhou China

**Keywords:** biofilm, *E. coli*, food additives, inhibition, *S. aureus*

## Abstract

The inhibition of microbial biofilms is a significant concern in food safety. In the present study, the inhibitory effect of sodium citrate and cinnamic aldehyde on biofilm formation at minimum inhibitory concentrations (MICs) and sub‐MICs was investigated for *Escherichia coli* O157:H7 and *Staphylococcus aureus*. The biofilm inhibition rate was measured to evaluate the effect of sodium citrate on *S. aureus* biofilms at 24, 48, 72, and 96 hr. According to the results, an antibiofilm effect was shown by both food additives, with 10 mg/ml of sodium citrate exhibiting the greatest inhibition of *S. aureus* biofilms at 24 hr (inhibition rate as high as 77.51%). These findings strongly suggest that sodium citrate exhibits a pronounced inhibitory effect on biofilm formation with great potential in the extension of food preservation and storage.

## 1 INTRODUCTION

Enterohemorrhagic *Escherichia coli* (EHEC) O157:H7 and *Staphylococcus aureus* are major pathogens associated with foodborne illnesses worldwide. EHEC causes bloody diarrhea and potentially life‐threatening hemolytic uremic syndrome by colonizing the intestine (Slayton et al., 2013), and virulent *S. aureus* is a leading nosocomial pathogen with a high occurrence rate; patients with implanted medical devices are particularly at risk for chronic staphylococcal infection (Authority, 2015). Pathogenesis, virulence and persistence of such pathogens are well documented to be associated with biofilm formation (Liu, Chen, et al., 2016), as antibiotics abused in animal and plant production have been transferred to humans by the food chain (Ter Kuile, Kraupner, & Brul, 2016); however, bacterial biofilms are more resistant to conventional antibiotics and host defences than the cells in suspension and contribute to bacterial persistence in chronic infections (Uhlich, Rogers, & Mosier, 2010). Therefore, biofilm formation by EHEC and *S. aureus* is regarded as a major issue because these biofilms exhibit increased resistance to antimicrobial agents in the food industry (Oloketuyi & Khan, 2017a; Srey, 2013).

Bacterial biofilms are formed when unicellular organisms aggregate to form a community that is attached to a solid surface (such as polystyrene, glass, and stainless steel in different environments) and encased in exopolysaccharide matrix, proteins, lipids, and DNA (Costerton, 1999; Liu, Deng, et al., 2016; Liu, Zhou, et al., 2016). Microbial biofilms are highly tolerant to salt concentration, desiccation, heat, antibiotics, and other food antiseptics in the natural environment (Davies, 2003). Previous studies have also reported that microorganisms can increase the resistance to mechanical damage thousands of times by forming a complex biofilm structure (Dong, 2014; Guo et al., 2014; Huang, He, & Liew, 2015; Li, Wu, Li, Zhang, & Yu, 2017; Nishimura, Tsurumoto, Yonekura, Adachi, & Shindo, 2006; Ying, Mao, Liu, Wong, & Wang, 2016). The attachment of foodborne pathogens to surfaces during food processing and storage can easily cause cross contamination (Kumar & Anand, 1998). Recent studies have suggested that the separation and enrichment of antibiofilm ingredients from burdock leaves could significantly improve biofilm inhibition (Lou, Song, Hong, Wang, & Lin, 2013). However, the high expense of this process remains a major obstacle. In addition, a number of food additives, such as nitrite (Steffen, Christiane, Birkenstock, Florian, & Friedrich, 2007), citric acid (Akbas & Kokumer, 2015), and cinnamic aldehyde (Oloketuyi & Khan, 2017b), have been found to exhibit antimicrobial effects against both gram‐positive and gram‐negative bacteria. Hence, the utilization of physical or chemical methods can negatively affect food quality and cause threats to human health as well as form biofilms on surfaces in contact with food. This hazard remains when using traditional food additives instead of physical or chemical methods (Simões, 2010). Compared with other antimicrobial agents, sodium citrate and cinnamic aldehyde extracted from natural plants have shown antimicrobial effects against foodborne pathogens (Sallam, 2007).

The goal of this study was to investigate the antibiofilm effects of sodium citrate and cinnamic aldehyde against *E. coli* O157:H7 and *S. aureus*, using methods including biofilm formation ability assays, microscopic analyses, biofilm inhibition tests, and optimization of biofilm inhibitor dose and reaction time.

## 2 MATERIALS AND METHODS

### 2.1 Bacterial strain preparation

*E. coli* O157:H7 (ATCC 43895) and *S. aureus* (ATCC 19095) were kindly provided by the Food Science and Engineering Microbiology Laboratory of Wuhan Institute of Technology. *S. aureus* and *E. coli* O157:H7 strains were inoculated on LB agar medium by the parallel scribing method and cultured in a 37℃ incubator for 24 hr. Single colonies that grew well on the LB agar medium were picked and inoculated into LB liquid medium and cultured in a 37℃ incubator for 24 hr, followed by serial dilutions using LB liquid medium to a target concentration (~10^6^ CFU/ml). The activated bacterial suspension was placed at 4℃ for further use.

### 2.2 Detection of biofilm formation ability

Overnight cultures of *E. coli* O157:H7 (50 µl) and *S. aureus* (50 µl) were individually transferred to Congo red medium (Sinopharm chemical reagent Co., Ltd. Shanghai, China.) and incubated overnight at 37℃ to observe the growth of colonies over 24 hr and 48 hr.

### 2.3 Biofilm formation in 12‐well microtiter plates

Biofilm formation was inspected as described previously (Cassat, Lee, & Smeltzer, 2007). Aliquots of 2% agar solution (1 ml) and sterilized coverslips (18 mm × 18 mm) were added to 12‐well microtiter plates. After the agar was completely set and the coverslip was fixed, a 3 ml aliquots of the suspension was transferred to each of three wells in a 12‐well microtiter plate and incubated at 37℃ for 7 days. The biofilm samples were washed with sterile PBS to remove unattached cells, and the biofilm cells were removed by swabbing with sterile cotton swabs. The swabs were transferred to 100 ml of physiological saline shaken vigorously and enumerated by standard spread‐plate technique (Joseph, Otta, Karunasagar, & Karunasagar, 2001). In addition, the coverslips were gently washed three times with phosphate‐buffered saline (PBS; pH 7.4; 8.0 g/L NaCl, 0.2 g/L KCl, 1.42 g/L Na_2_HPO_4_ 7H_2_O, and 0.27 g/L NaH_2_PO_4_) and stained with 1% crystal violet (Sinopharm chemical reagent Co., Ltd. Shanghai, China) for 15 min at room temperature (25℃). Extra crystal violet was eliminated by washing with normal saline solution three times and then the coverslips resuspended in 95% alcohol solution. Finally, the morphology of the biofilm was observed under a microscope at 400 × magnification. The biofilm was quantified by measuring the corresponding OD_620 nm_ with a UV‐visible spectrophotometer. Experiments were performed in triplicate for each strain, and the mean absorbance value was regarded as reflecting the biofilm formation ability of the strains.

### 2.4 Minimum inhibitory concentration (MIC) determination

The minimum inhibitory concentration (MIC) and minimum bactericidal concentration (MBC) of *S. aureus* and *E. coli* O157:H7 were separately determined using the double gradient dilution method (CLSI, 2015); namely, the sodium citrate and cinnamic aldehyde solutions were diluted twice using LB broth based on the application amount for food preservation (CLSI, 2015). Aliquots of the double‐diluted food additives (100 µl) and the bacterial suspension (100 µl) were added to each well in 96‐well microtiter plates. Simultaneously, 200 µl of bacterial suspension only was used as the negative control group, and LB broth was set as a blank control group. Finally, the samples were incubated at 37℃ for 24 hr. The MICs of the two food additives against *S. aureus* strains were determined by observing the turbidity of the solution. With regard to *E. coli* strains, the MICs of the two food additives were examined by measuring the relevant OD_620 nm_. The MBC was defined as the lowest concentration without colony growth on LB agar medium after 24 hr incubation at 37℃.

### 2.5 Effect of sub‐MICs of two types of food additives on the survivability of test bacteria

The effect of two types of food additive sub‐MICs on the survivability of *S. aureus* and *E. coli* O157:H7 was evaluated utilizing a growth curve analysis. In brief, a loop of the foodborne pathogen after culture with the corresponding concentration of food additive (8 MIC, 4 MIC, 2 MIC, MIC, 1/2 MIC, 1/4 MIC, and 1/8 MIC) was inoculated into 100 µl of LB broth and incubated at 37℃ for 24 hr. The OD (600 nm) of cultures was monitored using a UV‐visible spectrophotometer. The sub‐MIC used was half of the lowest MIC value, whenever only one food additive was added to the bacterial cell suspension, and one‐fourth of the MIC value, whenever combinations of the two food additives were added to the bacterial cell suspension. These concentrations were not high enough to inhibit bacterial growth, except in a few specific cases of synergism. Concisely, the cultured biofilms were incubated in 96‐well microtiter plates including aliquots of the double‐diluted food additives (1 ml) and LB broth (1 ml) at 37℃ for 2 days. The supernatant of the wells was absorbed with a rubber head dropper and gently washed with PBS. The biomaterial was fixed with methanol (200 µl) for 20 min at room temperature. After methanol was removed, the samples were stained with 1% crystal violet for 15 min and washed twice with PBS. After the sample was dried at room temperature, 33% glacial acetic acid (200 µl) was added to each well to dissolve the stained biofilm, and the OD_620 nm_ of the cultures was monitored every 24 hr up to 96 hr using a UV‐visible spectrophotometer. We made the following additions for *S. aureus* strains. Biofilms on the coverslips were lightly washed three times with PBS and stained with 1% crystal violet for 15 min at room temperature (25℃). Excess crystal violet was eliminated by washing with normal saline solution three times and then resuspended in 95% alcohol solution. Finally, the morphology of the biofilm was observed under a microscope at 400 × magnification.

#### 2.5.1 Relationship between the food additive biofilm inhibition effect and time

First, biofilms were cultured at 37℃ for 24, 48, 72, and 96 hr. Second, food additives (100 µl) of sub‐MIC concentrations that could inhibit biofilm formation were added to 96‐well microtiter plates including biofilms formed at 24, 48, 72, and 96 hr. Finally, the biofilms were detected as described in section 2.32.3 of the materials and methods.

The biofilm inhibition rate was calculated by referencing the following formation (Maunel, Lucia, & Maria, 2009):

$$\text{inhibition rate}\left(\%\right)=\frac{OD_{\mathrm{control}\mathrm{group}}-OD_{\mathrm{processing}\mathrm{group}}}{OD_{\mathrm{control}\mathrm{group}}}$$

### 2.6 Statistical analysis

All tests were performed in triplicate, the data gained from the experiments are shown as average values, and the distinctive points among the experimental data were illustrated and analyzed using the Origin Statistical Package, version 8.0.

## 3 RESULTS

### 3.1 The biofilm formation ability of E. coli and S. aureus strains

Significant changes were found in the region where Congo red medium was coated with colonies that turned from red to black after incubation for 48 hr, which demonstrated that the *E. coli* and *S. aureus* strains were capable of forming biofilms.

### 3.2 Biofilm formation and growth trends of test bacteria

The biofilm formation of the *E. coli* and *S. aureus* strains on coverslips was identified. In brief, the bacteria were smeared on LB agar medium with a cotton swab, and large colonies were observed. In addition, red *E. coli* O157:H7 colonies were formed in *E. coli* selective medium. This result showed that biofilms were formed on the coverslip surfaces.

**Figure 1:**
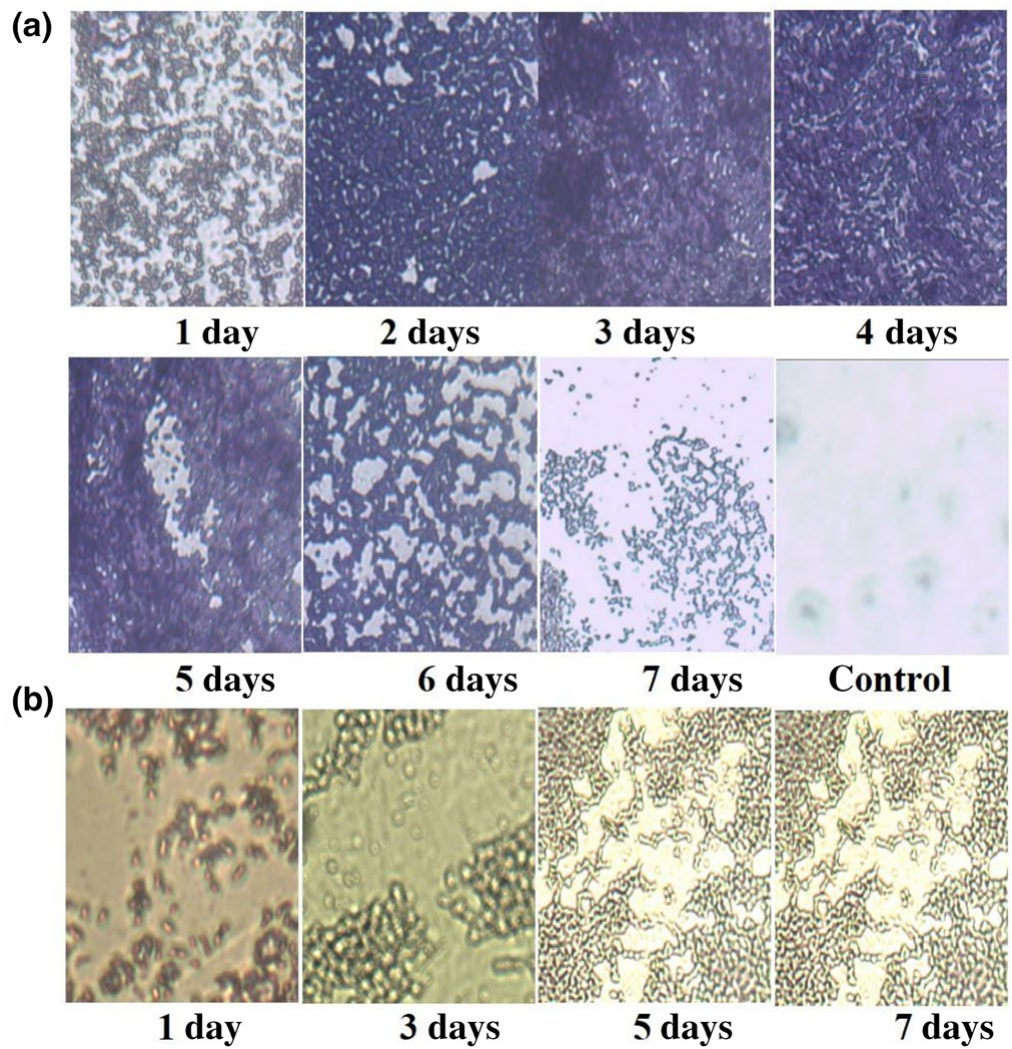
The growth trends of biofilm from the first day to the seventh day were observed via using crystal violet staining in optical microscope. (a) S. aureus strains. (b) E. coli strains

The growth trends of the test bacteria were studied using crystal violet staining and optical microscopy (Figure 1). With regard to *S. aureus* strains, the amount of biofilm increased from 1 to 4 days (Figure 1a). *S. aureus* was attached to the coverslip on the first day forming a loose mesh structure that was stained purple with crystal violet, which could be used to determine the adhesion of *S. aureus* strains on the coverslip and biofilm formation. The amount of biofilm continued to increase from the second day to the third day during the maturation of the biofilm. The growth rate was slower in the third and fourth days. The biofilm began to shed and decrease slowly from the fifth day, the overlapping area gradually disappeared, and the color gradually faded away. The least amount of biofilm formation during the observation period was observed from this point up to the seventh day.

For the *E. coli* strain (Figure 1b), purple‐red rod‐like structures of single *E. coli* colonies was noticed in the first day, the distribution of individual colonies was more dispersed than that of *S. aureus*, and there was no aggregation or film formation. A small proportion of *E. coli* cells cultivated for 3 days gradually aggregated but most maintained a similar morphology with single colonies. Most of the *E. coli* O157:H7 cultured for 5 days showed a sticky film‐like appearance, and only a few cells appeared in the free state. The *E. coli* strains cultured for 7 days completely aggregated to form a membranous structure. That is, the bacteria gradually accumulated to form a biofilm over time. Therefore, the area of the membrane gradually increased as the number of days of cultivation increased.

### 3.3 MIC and MBC determination

**Figure 2:**
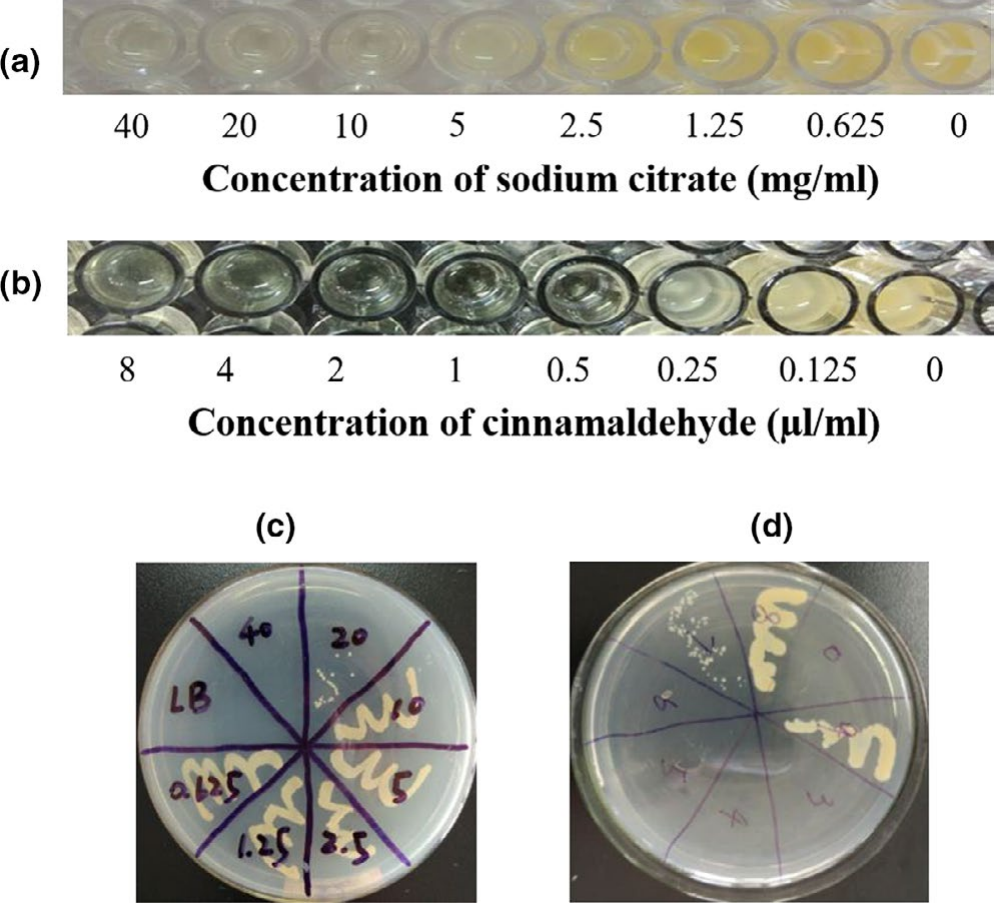
Minimum inhibitory concentration (MIC) and minimum bactericidal concentration (MBC) among S. aureus strains were determined using the double gradient dilution method, Observing the turbidity of solution and coating on LB agar medium. (a) Sodium citrate solution and (b) Cinnamic aldehyde solution for inhibition of biofilm in S. aureus strains. The growth of S. aureus colonies on the LB medium is shown shows the sterilization ability under different food additives concentration‐ (c) Sodium citrate solution and (d) Cinnamic aldehyde solution

The minimum inhibitory concentrations (MICs) of two types of food additives were determined against biofilm‐forming strains of *E. coli* and *S. aureus*. The results of the MIC determination in *S. aureus* were obtained by observing the turbidity of the culture solution (Figure 2a,b). The MICs of sodium citrate and cinnamaldehyde against *S. aureus* were 5 mg/ml and 0.5 µl/ml, respectively. As shown in Figure 2c,d), the minimum bactericidal concentration (MBC) against *S. aureus* was 40 mg/ml; similarly, the MBC of cinnamic aldehyde was determined to be 2 µl/ml.

**Figure 3:**
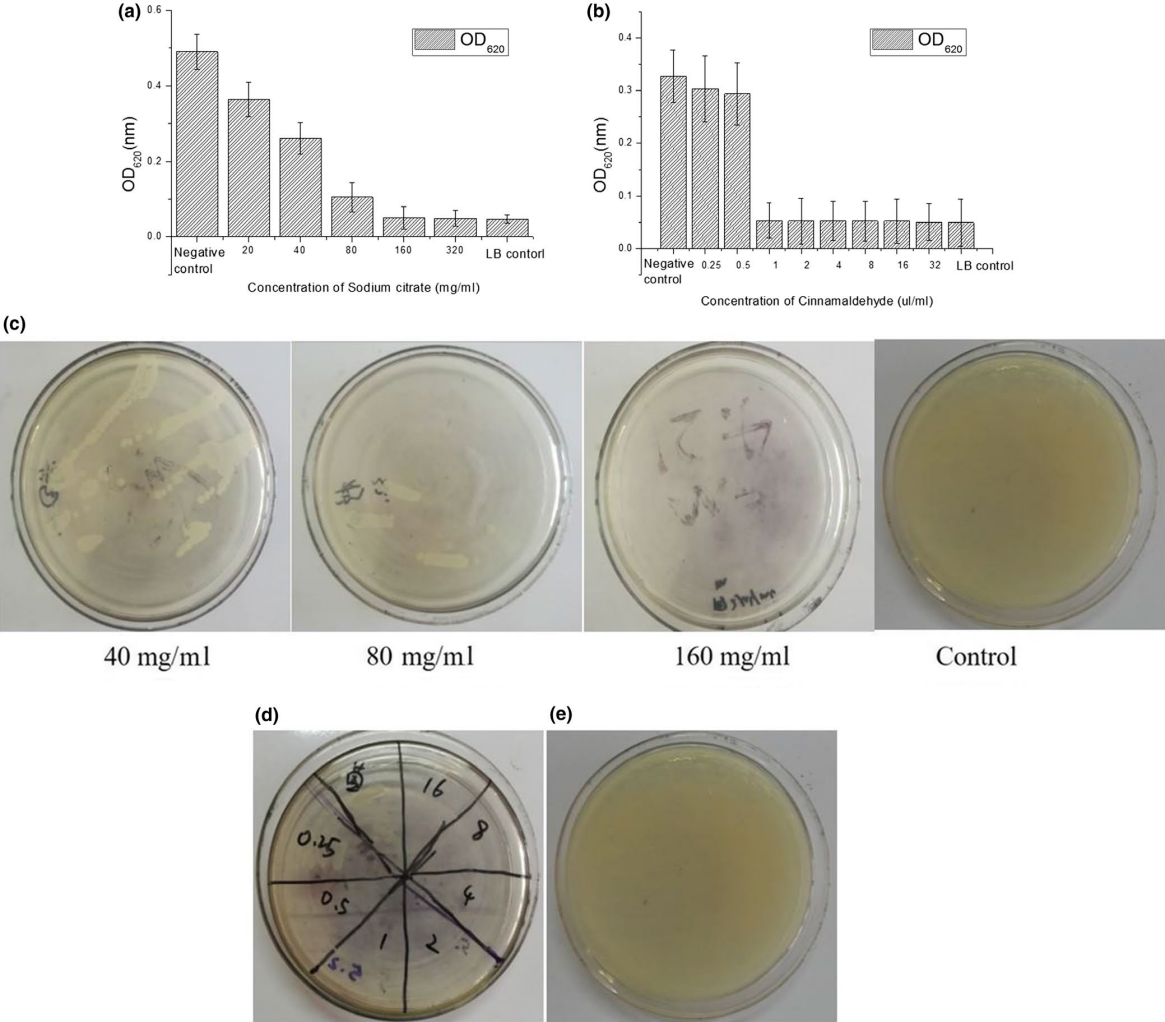
MIC and MBC among E. coli strains were assessed using growth curve analysis. (a) Sodium citrate solution and (b) Cinnamic aldehyde solution for inhibition of biofilm in E. coli strains. The data represents mean values of three independent experiments. Bars represent the mean ± standard error. The growth of E. coli colonies on the LB medium is shown shows the sterilization ability under different food additives concentration‐ (c) Sodium citrate solution and (d) Cinnamic aldehyde solution

The MIC and MBC of the food additives against *E. coli* were determined by combining the OD_620 nm_ value and the growth of colonies in the culture medium. There was no antibacterial effect when the sodium citrate concentration was less than 20 mg/ml (Figure 3a). With the increase of sodium citrate concentration, the measured OD (620 nm) values also decreased gradually. After the concentration of sodium citrate was increased to 160 mg/ml, the OD (600 nm) values were no longer diminished and tended to be stable. Consequently, the MIC of sodium citrate is approximately 160 mg/ml. As shown in Figure 3b, a small amount of bacteria grew at 40 mg/ml and 80 mg/ml, respectively. *E. coli* cells did not grow in the medium with 160 mg/ml sodium citrate. The microplate reader‐measured data analysis showed that the MBC of sodium citrate is 160 mg/ml (a comparison with references showed that the data are within reasonable limits). Similarly, the analysis of Figure 3a–e) shows that the MIC and MBC of cinnamaldehyde against *E. coli* biofilms is 1 µl/ml.

### 3.4 The microscopy results of S. aureus biofilm inhibition by sodium citrate

**Figure 4:**
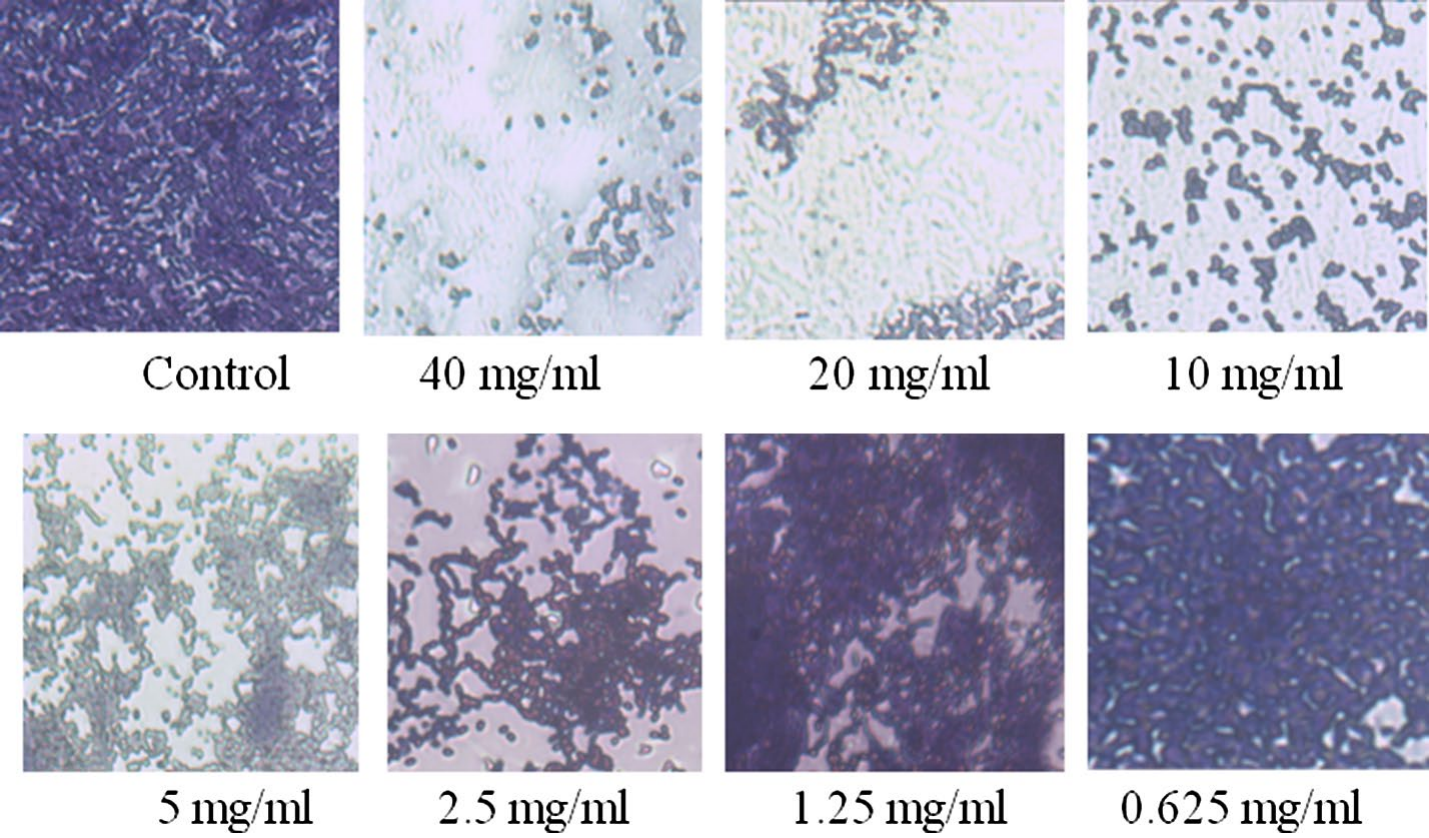
Microscopic examination of biofilms with different concentrations of sodium citrate among S. aureus strains

The *S. aureus* biofilm inhibition by sodium citrate findings are depicted in Figure 4. As the concentration of sodium citrate increased, the quantity of the biofilm formed by *S. aureus* strains decreased by degrees. The formed biofilms became increasingly sparse in response to concentrations of sodium citrate ranging from 0.625 mg/ml to 40 mg/ml, which indicated that sodium citrate treatment had a significant negative effect on the *S. aureus* biofilm compared with the untreated control. At the sodium citrate concentration of 40 mg/ml, the biofilm formation was reduced to the greatest extent. In contrast, at the sodium citrate concentration of 0.625 mg/ml, there was almost no inhibitory effect on the amount of biofilm, which was the same as the control group without added sodium citrate. In addition, when the sodium citrate concentration increased from 0.625 to 20 mg/ml against the test pathogens, the crystal violet color of the biofilm gradually decreased, and the overfill area decreased continuously, indicating that the inhibitory effect of sodium citrate on the biofilm gradually increased.

### 3.5 Effect of sub‐MICs of two types of food additives on the survivability of test bacteria

**Figure 5:**
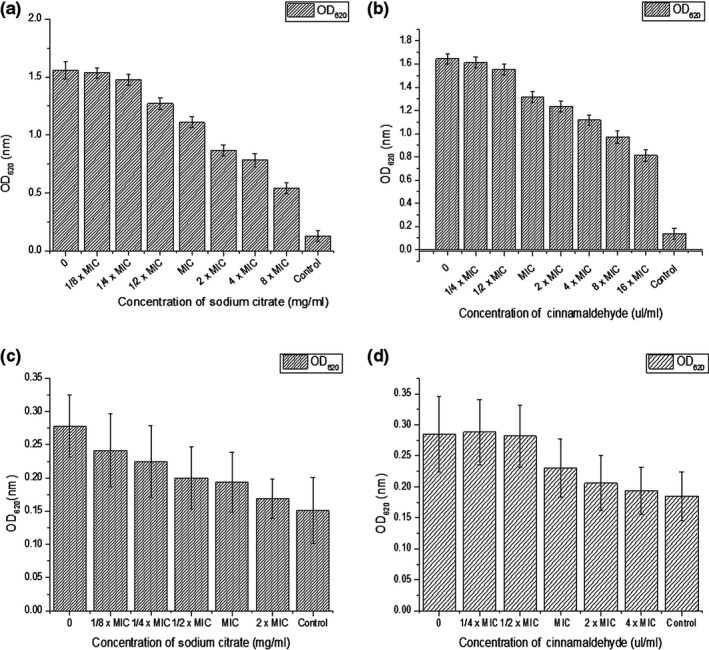
Inhibition of biofilm formation by sub‐inhibitory concentrations of two food additives in S. aureus strains and E. coli strains. (a) Sub‐inhibitory concentrations of sodium citrate solution for inhibition of biofilm in S. aureus strains. (b) Sub‐inhibitory concentrations of cinnamic aldehyde solution for inhibition of biofilm in S. aureus strains. (c) Sub‐inhibitory concentrations of sodium citrate solution for inhibition of biofilm in E. coli strains. (d) Sub‐inhibitory concentrations of cinnamic aldehyde solution for inhibition of biofilm in E. coli strains. The data represents mean values of three independent experiments. Bars represent the mean ± standard error. It shows significantly different at p =0.05

The sub‐MIC treatment effect of two types of food additives on biofilms is depicted in Figure 5a–d; the treatments displayed a concentration‐dependent reduction of *E. coli* and *S. aureus* biofilm formation. Similarly, treatment with sub‐MICs of sodium citrate (1/8 × MIC‐4 × MIC) demonstrated that sodium citrate showed 1.54%, 5.32%, 18.5%, 28.93%, 44.58%, and 49.78% reductions in biofilm biomass at sub‐MICs. Furthermore, *S. aureus* strains exhibited 2.07%, 5.65%, 20.11%, 24.97%, 32.2%, 40.95%, and 50.73% inhibition rates in biofilm biomass in the 1/4 × MIC‐16 × MIC range of cinnamaldehyde. These results suggest that the inhibition capability of sodium citrate against *S. aureus* is stronger than that of cinnamaldehyde at the equivalent sub‐MIC. For *E. coli*, treatment with sub‐MICs of sodium citrate (1/8 × MIC‐2 × MIC) showed 13.09%, 19.06%, 18.5%, 28.06%, 30.21%, and 39.21% reductions in biofilm biomass (Figure 5c). *E. coli* strains displayed 1.05%, 1.05%, 19.30%, 27.72%, and 32.28% inhibition rates in biofilm biomass in the 1/4 × MIC‐4 × MIC range of cinnamaldehyde (Figure 5d). The comparative analysis of two types of food additives showed that the inhibition capability of sodium citrate was stronger than cinnamaldehyde at the equivalent sub‐MICs in *E. coli*. In contrast, since sodium citrate had the best inhibitory effect on the biofilm production by *S. aureus*, we next explored the relationship between the inhibition effect and time in *S. aureus*.

### 3.6 The relationship between the biofilm inhibition effect of sodium citrate and time

**Figure 6:**
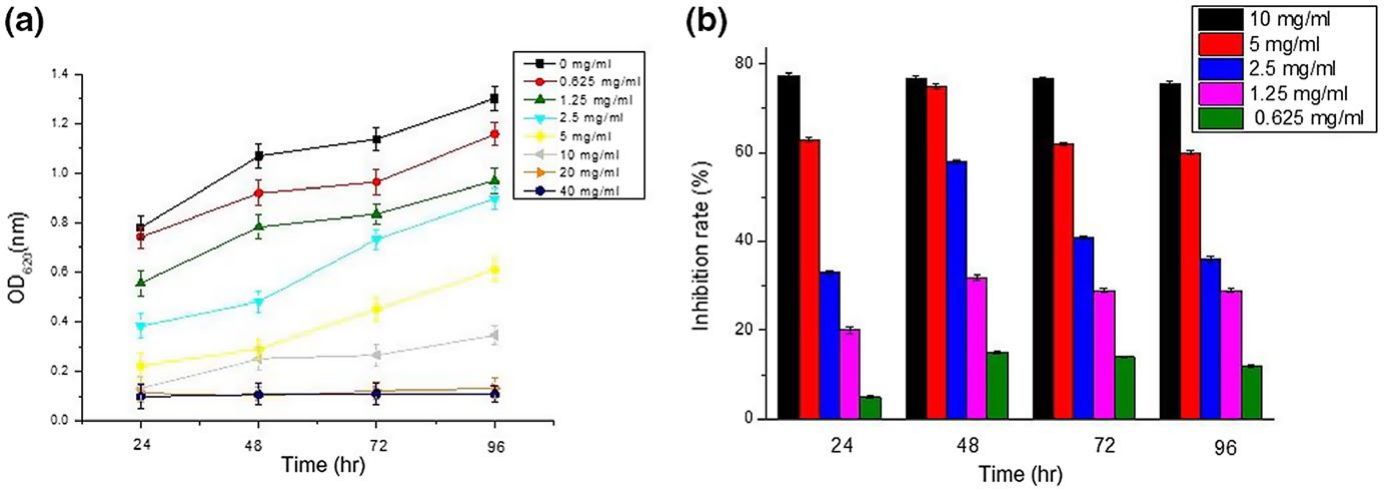
Relationship between sodium citrate suppressive effect and time in S. aureus. (a) The OD (600 nm) under different concentrations of sodium citrate solution was separately measured at 24 h, 48 h, 72 h, 96 h. (b) The inhibition rate trend of 0.625‐10 mg/ml of sodium citrate solutions on the biofilm have been shown at 24 h, 48 h, 72 h, 96 h. The data represents mean values of three independent experiments. Bars represent the mean ± standard error

The effect of sodium citrate treatment on biofilm formation at 24 hr, 48 hr, 72 hr, and 96 hr is depicted in Figure 6a. When the concentration of sodium citrate ranged from 0.625 to 10 mg/ml, the amount of biofilm increased over time up to 96 hr. At sodium citrate concentrations of 0.625 mg/ml and 1.25 mg/ml, the increasing trends of biofilm formation ranging from 48 hr to 96 hr was almost the same as the samples containing only bacteria (0 mg/ml sodium citrate), which indicated that the effect of inhibition on *S. aureus* biofilms was weakened at 0.625 mg/ml and 1.25 mg/ml of sodium citrate. As the concentration of sodium citrate increased to 20 mg/ml, *S. aureus* biofilm formation was markedly inhibited because the concentration of bacteria was not sufficient to form a membrane. Moreover, the OD_620 nm_ values were close to the control group, including only the LB liquid medium. When the concentration of sodium citrate was increased to 40 mg/ml, *S. aureus* bacteria were almost completely killed before forming biofilms. The OD_620 nm_ values changed slightly during the cultivation periods, and biofilm formation was not observed.

The biofilm inhibition rate was calculated at the concentration of sodium citrate ranging from 0.625 to 10 mg/ml against *S. aureus* because *S. aureus* did not form biofilms at sodium citrate concentrations of 40 mg/ml and 20 mg/ml. As shown in Figure 6b, the inhibition rate of the concentration from 0.625 mg/ml to 5 mg/ml initially increased and then decreased within 24–96 hr. However, the biofilm inhibition rate was more than 75% at 10 mg/ml of sodium citrate within 24–96 hr. Therefore, the maximum inhibition rate of biofilm reached 77.51% at 24 hr in *S. aureus*.

## 4 DISCUSSION

Biofilms have been considered to account for 80% of all microbial infections or contaminations during food processing (Antonio, Maria, & Milena, 2017), and consequently, inhibition of biofilms by natural or potentially edible substances is important. In recent years, biocompatible poly (lactic‐co‐glycolic acid, such as essential oils) coatings containing clove oil or eugenol have been found to exhibit efficient biofilm inhibition on solid surfaces and attenuate the virulence of *E. coli* (Kim et al., 2016). Additionally, a ubiquitous oligosaccharide in human milk, 1‐Amino‐2′‐fucosyllactose (2′‐FL), has been reported to be an antibacterial agent against group B Streptococcus (GBS) as conversion of 2′‐FL to its reducing end product β‐amine provided a novel antibiofilm compound despite being devoid of any substantial antimicrobial or antibiofilm activity (Craft & Townsend, 2019). In addition, *N*‐benzyl anilines as inhibitors of fatty acid synthesis have been studied, and compound 4k was found to exhibit strong antibacterial activity against clinical MRSA strains and inhibits biofilm formation by in vitro biofilm inhibition and microscopy assays (Zhang et al., 2019). Recently, several studies have reported that ε‐polylysine in combination with nisin under acidic conditions shows increased inhibition of the growth of microorganisms (Waal et al., 2012); the mechanism is mainly due to the influence of electrostatic interactions and the difference in the ability to enter the biofilm. The inhibition of biofilms is influenced by the bivalent cations in the surrounding liquid; the positive charge which decreases the pH value and increases the ε‐polylysine concentration can effectively inhibit the formation of the biofilm (Maunel et al., 2009). Sybiya et al. (La, Agilandeswari, Musthafa, Karutha, & Veera, 2012) demonstrated that biofilm formation by gram‐negative bacterial pathogens treated with 10 µg/ml methyl eugenol was retarded when compared with that of untreated bacteria. The results of the biofilm inhibition test also support the observation of Zhang et al. (2014), who demonstrated a considerable reduction in the biofilm of foodborne pathogens (*Staphylococcus aureus* and *Salmonella enteritidis*). In other studies, a wide variety of natural compounds, such as eugenol, geraniol, and tea polyphenols, have also been reported to inhibit biofilm formation by foodborne pathogens (Husain, 2014; Zhang et al., 2014). Therefore, food additives with antibiofilm effects are highly required for food safety.

In this study, *S. aureus* and *E. coli* with strong biofilm‐forming abilities, as demonstrated by the Congo red plate method, were selected for the assessment of the antibiofilm effect of sodium citrate and cinnamic aldehyde. The biofilm inhibition test showed that at sub‐inhibitory concentrations, sodium citrate, and cinnamic aldehyde could inhibit the biofilm formation by both tested bacteria in a dose‐dependent manner, which is consistent with the results showing that the addition of cinnamaldehyde reduced the biofilm of *P. fluorescens* adhering to slides (Kim, Lee, Kim, Baek, & Lee, 2015; Li et al., 2018). Biofilm formation was not completely inhibited at 1/2 MIC and MIC of sodium citrate treatment in *S. aureus*, indicating that high concentrations of sodium citrate mainly reduced the number of bacteria by inhibiting bacterial growth to reduce the production of biofilm. In the present investigation, the greatest inhibition of *S. aureus* biofilm formation (77.51%) was found at a sodium citrate concentration of 10 mg/ml at 24 hr (Figure 6b). Our findings are similar to those of Friedman et al., who found cinnamaldehyde to be harmless in cosmetics, food, and hygiene products; up to 0.03% cinnamaldehyde is allowed in foods (Friedman, Kozukue, & Harden, 2000). Similarly, increased prevention and removal of biofilms and higher numbers of prevented or removed *S. aureus* strains have been shown for citric acid (2% or 10%, w/v) treatments compared to peracetic acid treatments (0.3%, v/v) (Akbas & Kokumer, 2015). Therefore, it is assumed that sodium citrate, cinnamic aldehyde, and other food additives derived from natural products can be used as strategies to control biofilm formation by foodborne pathogens.

## 5 CONCLUSION

In this study, the results obtained indicate a good ability of *S. aureus* and *E. coli* strains to form biofilms, which was validated by the Congo red plate method.

The growth of test bacteria over 7 days was observed via crystal violet staining. After the MIC was determined, sub‐MICs of two types of additives were used in the experiments and showed that the consequent reduction of biofilms was not due to growth inhibition. Finally, we explored the relationship between the biofilm inhibition effect of sodium citrate and time in *S. aureus*. From the results, we show that the maximum biofilm inhibition reached 77.51% at 24 hr in *S. aureus*. Although the mechanism of inhibition of biofilm formation by two types of additives is not fully understood, our study demonstrates the potential utilization of food additives derived from natural origins as prospective antibiofilm agents.

## CONFLICT OF INTERESTS

The authors declare no competing of interest.

## AUTHOR CONTRIBUTIONS

The research structure was conceived and designed by L.L., Z.X., and M.E.S.; C.Y. prepared the sample; W.X. and Y.M. performed the experiments. R.P. and J.C. analyzed the data and J.M.H., T.S. wrote the paper; X.Z. and J.X. made revision to the final manuscript. The final manuscript was read and corrected by all authors.

## ETHICS STATEMENT

None required.

## Data Availability

All data generated or analysed during this study are included in this published article.
